# Diffractive Efficiency Optimization in Metasurface Design via Electromagnetic Coupling Compensation

**DOI:** 10.3390/ma12071005

**Published:** 2019-03-27

**Authors:** Yang Li, Minghui Hong

**Affiliations:** Department of Electrical and Computer Engineering, National University of Singapore, 4 Engineering Drive 3, Singapore 117576, Singapore; eleliy@nus.edu.sg

**Keywords:** metasurface, coupling compensation, diffractive efficiency

## Abstract

Metasurface is an advanced flat optical component that can flexibly manipulate the electromagnetic wave in an ultrathin dimension. However, electromagnetic coupling among neighbored optical elements decreases the diffractive efficiency and increases the noise. In this paper, a novel computational method is proposed to optimize the coupling of the metasurface. The coupled electric fields in metasurface design are decomposed into various coupling orders and then restructured to replace the whole metasurface simulation. This method is applied to optimize a metasurface that consisted of conventional nanorod plasmonic antennas as a case study. The convergence of this method in calculation is demonstrated. The electric field intensity deviation of a nanoantenna array can be reduced from 112.2% to 0.5% by the second-order coupling correction. The diffractive efficiency of a three-level phase meta-deflector is optimized from 73% to 86% by optimized coupling compensation via particle swarm optimization (PSO). This process opens a new area of metasurface design by the detailed field distribution of optical elements.

## 1. Introduction

The electromagnetic couplings are important phenomena. They exist in many fields, including mutual inductance [1], plasmonics [2], and metamaterials [3]. In modern electromagnetic research, the coupling can lead to many unique effects, such as extraordinary optical transmission (EOT), negative refractive index, and high chirality. For the periodic design, the electromagnetic wave can transmit through subwavelength hole arrays on an opaque film. This EOT effect is due to the coupling of surface plasmons. However, for the non-periodic designs, too many parameters are involved in the design, which takes a long time in calculations for a complete scan of parameter optimization.

Metasurface is one of the most important optical designs involving non-periodic structures. It is an advanced flat optical device that consists of a large number of subwavelength optical antennas. In the development of a metasurface, the dimensions of the optical antennas become smaller, while more dielectric metasurfaces are designed, instead of metal metasurfaces [4]. These designs are applied for high efficiency and resolution light manipulation. On the other hand, it leads to weaker field localization. Thus, the performance of metasurface is obviously affected by electromagnetic coupling. To avoid this negative effect, a high refractive index of loss-free materials is desired for better field localization. In the infrared region, silicon is an ideal material with a high refractive index and low loss. However, when the metasurface is used in the visible spectrum, only 75% diffraction efficiency can be achieved due to the energy loss of the material [5]. Most optical materials with a high refractive index are not transparent. In this light spectrum, most materials with a high refractive index have high loss [6]. Silicon nitride (n ≈ 2), which has 90% transmission in the visible region, was used to design a metasurface. However, its diffraction efficiency is only about 40% [7]. Lower refractive index materials, such as glass, are barely selected in metasurface design due to lower field localization capability.

Due to the low diffraction efficiency caused by electromagnetic coupling, a series of negative effects appear. For instance, in a meta-hologram based on geometric phase, the noise of the unmodulated light (the zero-order noise) can be designed to be completely filtered out by the wave plate and polarizer. However, in practical holographic applications, the zero-order noise along the transmission direction cannot be completely removed in most cases due to electromagnetic coupling [6,8]. The designed phase shift is deviated randomly by the coupled field from the neighbor optical antennas, which leads to noise along the beam without modulation. The off-axis illumination is applied to improve the signal-to-noise ratio (SNR) in meta-holography [9,10,11]. However, this is only a compromise. The coupling also affects the symmetry of optical systems [12], which limits metasurface applications in optical integration, optical communication, and spatial multiplexing meta-holograms. Hence, it faces great challenges in the coupling compensation.

To overcome this issue, a novel design method of the metasurface is proposed in this paper by considering electromagnetic coupling. The element of the metasurface is simulated separately through coupling a group of optical antennas, instead of the traditional periodic design. The multiple orders of the coupling are modeled and simulated by field decomposition. This method is rigorous in theory, and its convergence is demonstrated. It gives an opportunity to consider the coupling in metasurface design. Combining particle swarm optimization (PSO) and our coupling compensation method, the diffractive efficiency of a meta-deflector is increased by 13%. It is found that the detailed light field through a metasurface can also be designed by this optimization method to achieve extraordinary improvement.

## 2. Principles

In traditional optics, there are some fundamental physics problems, such as optical aberration and multiple reflections [13,14]. The optical aberration can be reduced by a combination of multiple lenses with different radii and concave/convex designs in geometric shapes. In the simple cases, the chromatic aberration is modeled by the Abbe number of the lens materials. However, in a more complex system with multiple optical aberrations, the commercial software, such as Zemax (Zemax LLC, Bellevue, WA, USA), must be used to optimize the design. Similarly, for subwavelength optical design, such as metamaterial and photonic crystal [15,16,17,18,19], the parameters of periodic structures can be globally optimized via numerical simulation. However, for the non-periodic design, such as that of a metasurface, there are too many parameters. The elements of metasurface, which are called optical antennas, can modify the phase, amplitude, and polarization of light on a subwavelength scale [9,20]. For a complex optical function, there are thousands of optical antennas, but a lack of an effective method.

Conventionally, the optical antennas are optimized by a periodic design. Then, these antennas are arranged for light manipulation based on the optical property in periodic condition. However, the electromagnetic coupling among optical antennas is ignored. The complexity of the electromagnetic coupling makes the designed light manipulation difficult to be accurately realized. It needs time-consuming computations for optimization. Without proper design optimization, most functions of metasurface cannot be realized with high diffractive efficiencies (DEs).

**Figure 1:**
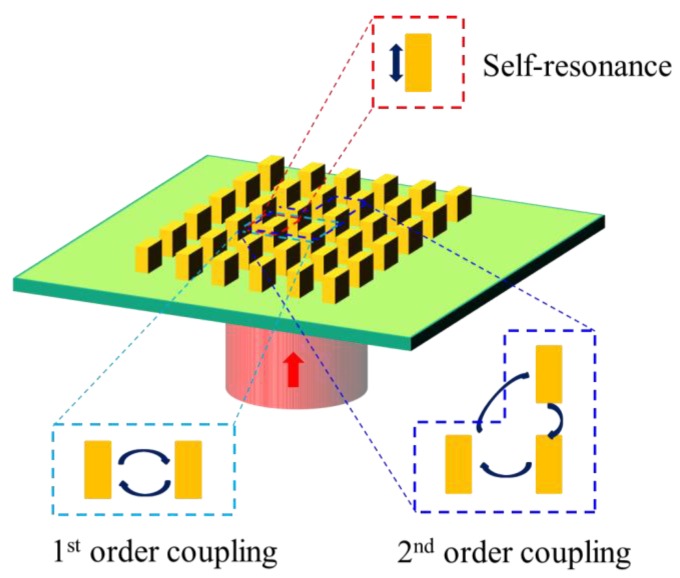
Schematic of the decomposition of electromagnetic coupling in metasurface design.

To overcome this issue, the fundamentals are the basic electric field distribution and the contribution of electromagnetic coupling. The electric field distribution of a metasurface at a plane parallel to the surface can be expressed as *E*_meta_(x,y) illuminated by a plane wave *E*_in_(x,y). The plane wave normally illuminates the metasurface. The *E*_in_ can be considered a constant. The *E*_meta_(x,y) can be decomposed into the electric field contributions of each optical antenna and the coupling among the antennas:

(1) $$\begin{array}{cc} E_{meta} & =E_{antenna}+E_{coupling}\\ & =\sum_{i}E_{antenna,i}+\sum_{i}E_{coupling,i}=\sum_{i}\left(E_{antenna,i}+E_{coupling,i}\right) \end{array}$$

where *E*_antenna, i_ and *E*_coupling, i_ are the radiated and coupled electric fields of the *i*th optical antenna. The electric field distribution of the whole metasurface can be restructured by summing the radiated and coupled electric fields of all the antennas together. The radiated electric field distribution of the *i*th antenna can be simulated by local illumination on a single antenna. It is difficult to calculate the coupling for each antenna, as all the optical antennas are different. Hence, the electric field induced by the coupling is further decomposed by the number of the coupled optical antennas.

(2) $$\begin{array}{cc} E_{0,i_{1},i_{2},\ldots ,i_{n}} & =E_{0}+\sum_{m_{1}=i_{1},i_{2},\ldots ,i_{n}}\Delta E_{0,m_{1}}+\sum_{m_{1},m_{2}=i_{1},i_{2},\ldots ,i_{n}}\Delta E_{0,m_{1},m_{2}}\\ & +\ldots +\sum_{m_{1},m_{2},\ldots ,m_{n}=i_{1},i_{2},\ldots ,i_{n}}\Delta E_{0,m_{1},m_{2},\ldots ,m_{n}}+\ldots \end{array}$$

where $\sum_{m_{1},m_{2},\ldots ,m_{k}=i_{1},i_{2},\ldots ,i_{n}}\Delta E_{0,m_{1},m_{2},\ldots ,m_{k}}$ is the *k*th order coupling. Figure 1 shows the schematic of the decomposition of the metasurface coupling. The radiated field of a single antenna is defined as *E*_0_, which is generated by the resonance of the optical antenna itself. The coupling between the illuminated antenna and one coupled antenna is defined as the first-order coupling correction; the coupling among the illuminated antenna and two coupled antennas is defined as the second-order coupling. The higher order coupling can be defined in the similar way. Conventionally, the higher order coupling is weaker due to the increasing spatial separation and limited radiation efficiency. The coupling can be corrected by the finite order of calculation. In this way, the coupling effects for all the optical antennas are similar. A database of all the coupled electric field distributions can be built for the compensation. The irradiated and coupled electric fields of each antenna can be directly calculated from the database. Hence, the light distribution can be calculated accurately without whole model simulation. The accuracy of this coupling compensation method will be discussed, and then it will be applied for DE optimization.

## 3. Results and Discussion

### 3.1. Coupling Compensation

**Figure 2:**
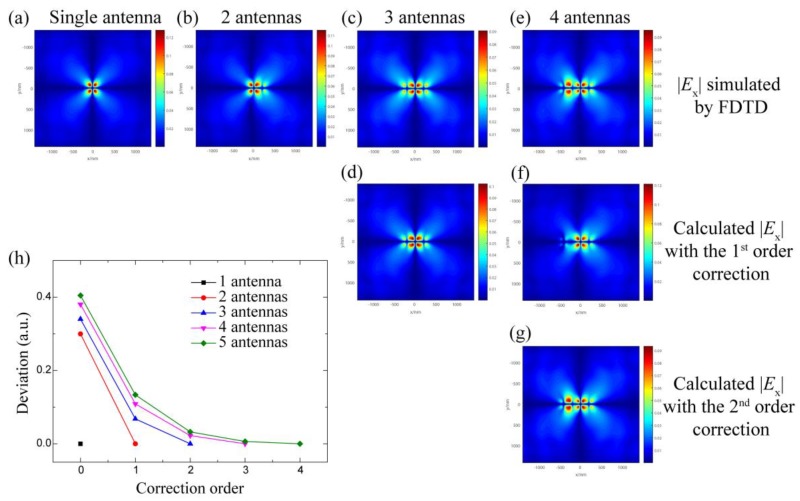
Simulated electrical filed distribution along x-axis (*E*x) distributions simulated by FDTD (**a**–**d**) and calculated by the field of the elements with the first-order (**e**,**f**) and second-order (**g**) corrections (**h**) Field deviation for various correction orders.

To prove that the above-proposed method can calculate the field distribution efficiently for practical applications, it is important to investigate the calculation convergence. A traditional gold nanorod antenna is chosen as a case study. The lattice, length, and width of optical antennas are 200 nm, 140 nm, and 60 nm, respectively. The polarization of the incident light is parallel to the nanorod. The illumination area is 200 nm × 200 nm at 100 nm below the metasurface at an incident light wavelength of 800 nm. The grid size is set as 10 nm. By numerical simulation with a finite-difference time-domain (FDTD) software (Lumerical Inc, Canada), the electric field distributions of single and multiple antennas are shown in Figure 2a–d. Although the incident light only illuminates the optical antenna located at (0, 0), the field distributions of the multiple antennas are different from the field distribution of a single antenna *E*_0_ (Figure 2a). The relative electric field deviation Δ*E*/*E*_0_ is as high as 30% to 40% without considering the coupling. Figure 2d,e show the restructured field distributions with the first-order and second-order coupling corrections. For a three-antenna system, the deviation can be decreased from 34.0% to 6.8% by the first-order coupling correction. For a four-antenna system, the deviation can be reduced to 10.9% and 2.25% by the first-order and second-order coupling corrections, respectively.

These results indicate that the coupling correction is efficient, rigorous, and convergent in a calculable system. Similar results can be observed for a system with more optical antennas, as shown in Figure 2h. The deviation decreases inversely proportional to the correction order. It can be explained by the multiple scattering of the coupled optical antenna. The electric field excited by the scattering field from a neighbored antenna can be assumed as *αE*_0_, where *α* is a coupling factor between zero and one. That is the first-order coupling. Considering the second-order coupling, the antenna with *αE*_0_ can be treated as a subsource to excite another neighbored antenna that the intensity of the second-order coupling can be estimated as *α*^2^*E*_0_. In the calculation, it is difficult to get the accurate value of *α* due to the complexity of the coupling. It also depends on the orientations of the optical antennas; the value is different for different order corrections. In this case, *α* is in the range of 0.2 to 0.4, which is smaller than one. It is convergent in the calculation of the coupling corrections.

**Figure 3:**
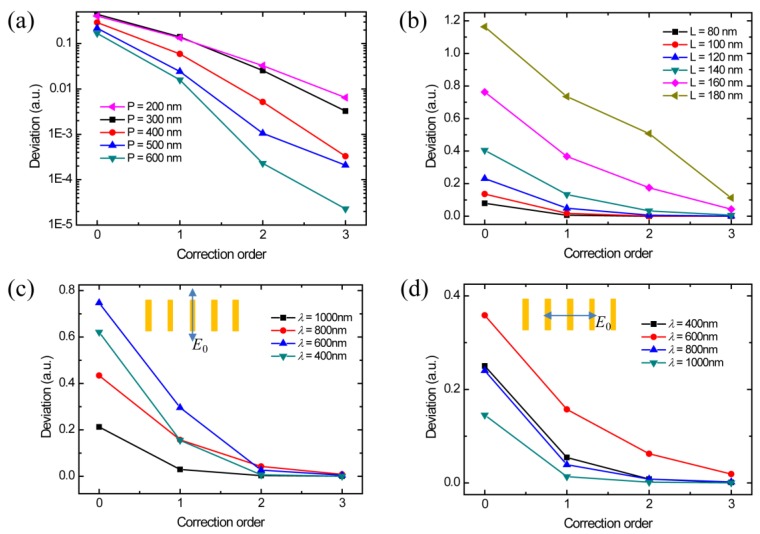
Calculated |*E*x| deviations compared with the simulated results at different (**a**) periods, (**b**) lengths of optical antenna, (**c**) wavelengths, and (**d**) polarizations.

The coupling factor is one important parameter to the convergence of the coupling correction. The calculation time and the accuracy need to be properly balanced. The coupling intensity depends on the geometric parameters of the structure design, wavelength, and polarization. In the above cases, the geometric parameters include the length, width, height, and period of optical antennas. The performance of such a metallic rod design is not sensitive to the width and height [21]. The period of the optical antenna directly affects the distance between two antennas. The coupling is strong at a short distance and weak as the distance increases. It is due to the electric field radiated by subsources, which can be estimated as inversely proportional to the square of the distance. The antenna is considered as a point source. Figure 3a demonstrates that the coupling intensity reduces when the period increases. At the periods of 200 nm and 300 nm, the coupling intensities are almost the same, which is attributed to the near-field coupling at a deep subwavelength distance. Theoretically, when the period of the antenna is large enough, the coupling does not need to be considered. However, the efficiency and the maximum controllable special frequency would be affected at a large period. To fully control the wave factors in free space, the period of the optical antenna cannot be designed to be larger than the wavelength. Therefore, the coupling in the metasurface design cannot be avoided. The length of the optical antenna is another factor affecting the coupling, which affects the antenna resonance. The length of the optical antenna is usually at deep subwavelength scale for the metasurface design, which is shorter than the resonant length in the dipole model. Therefore, the increasing of the length of the antenna leads to stronger resonance and also affects its coupling. As shown in Figure 3b, the coupling of longer nanorods is stronger so that a higher order of correction is required. Figure 3c,d show the deviation caused by the coupling at different wavelengths and polarizations for comparison. The highest deviation always happens at the wavelength around 600 nm, which is at the plasmonic frequency of gold. Since the deviation in both polarization illuminations can be corrected with good convergence, the light field of common optical antennas can be restructured by this new coupling correction method.

**Figure 4:**
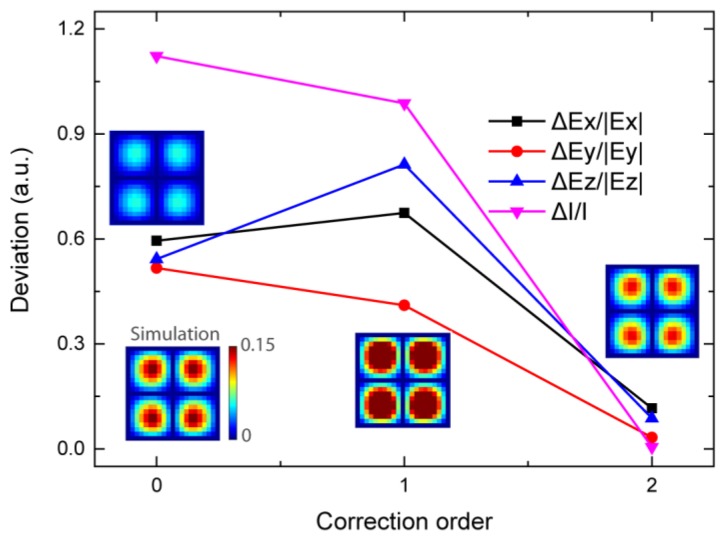
Average deviation of the restructured electric field at different order corrections. Insets: simulated and restructured electric field distributions |*E*_x_| of a periodic optical antenna array.

In a more complex system with a large number of antennas, it is impossible to calculate all the coupled electric fields for all the antennas. However, the deviation of the electric field can be greatly reduced by the coupling correction via our proposed method to calculate only a limited area. For instance, a periodic optical antenna array is simulated. As shown in the insets of Figure 4, the electrical fields indicate the distribution through numerical simulation and the restructured field distribution via the field superposition. The results indicate that the field distributions calculated by different methods are similar, but the intensity is greatly changed. Without the correction, the radiated field is very weak. Hence, the deviation is as high as 112.2%. With the first-order correction, the deviation is slightly reduced to 98.7%. After the second-order correction, the deviation of the calculated electric field intensity is rapidly reduced to 0.5%. The restructured distribution and intensity of light match the simulated results very well. It is obvious that the coupling correction with the limited orders is efficient for metasurface design with a large number of elements.

### 3.2. Diffractive Efficiency Optimization

The deflector based on the metasurface design is similar to the optical wedge and grating. One optical wedge can change the direction of the light without energy loss, which has a continuous linear phase profile to tune the wavefront. However, it is difficult to design and fabricate for a large deflection angle. The thickness of the high deflection angle wedge becomes large. Secondly, in diffractive optics, the grating can act as a deflector. Its diffractive efficiency is related to the number of phase levels. For a two-level grating, the DE is only about 40%, while the DE can achieve more than 90% for an eight-level grating. On the other hand, higher levels correspond to more complex procedures in fabrication.

Metasurface can provide a perfect solution theoretically. For an ideal case, the optical antenna can be designed with the scale much smaller than the wavelength. The designed metasurface with multiple phase levels can achieve a high DE. However, the optical antenna cannot be designed in a deep-subwavelength scale, which is limited by the optical properties of the materials in the visible and ultraviolet spectra. Therefore, the DE of the metasurface is not high enough due to the discontinuous phase profile. How to use the discrete optical antennas to control the continuous phase profile is a challenge in optical design.

To achieve a continuous phase profile, detail electric field distribution needs to be considered in the metasurface design. With this coupling decomposition method, it can be restructured via calculation after building a database of the detail radiated and coupled field distribution of optical antennas. The nanorod antenna is the most common phase modulator in the metasurface design due to its geometric phase effect to the cross-polarized light. The phase shift is twice the rotation angle without considering the coupling. To avoid time-consuming computation, the first-order coupling is chosen for diffractive efficiency optimization. The wavelength is changed to 400 nm, while the other parameters are the same. As shown in the simulation results in Figure 3c,d, the first-order coupling can significantly reduce the deviation. The electrical fields of optical antennas are calculated at a rotation step of 30 degrees. The electrical field distribution of the optical antenna with a more accurate rotation angle is calculated through linear interpolation. Only coupling from neighbored antennas is considered.

**Figure 5:**
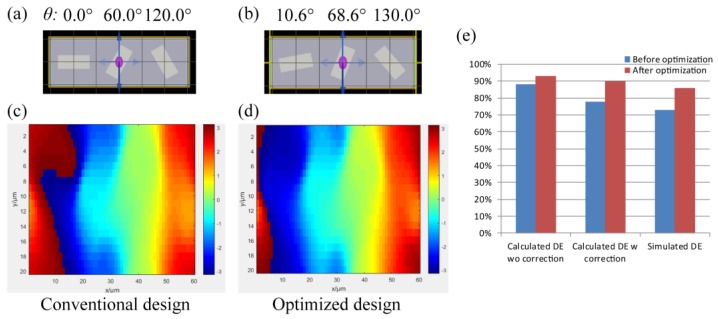
Schematics and simulated phase distributions of the *E*x of (**a**,**c**) the conventional design and (**b**,**d**) optimized design of a meta-deflector. (**e**) The diffraction efficiency improvement at the calculation with and without the correction and simulation.

For deflector-based three-level optical antennas, the rotation angles can be simply set as 0°, 60°, and 120° (Figure 5a), corresponding to the phases of 0, 2π/3, and 4π/3, respectively. The designed phases are discrete. Its DE can achieve 73%, which is higher than ~60% for the theoretical limits of three-level phase gratings. From the simulated phase distribution shown in Figure 5c, it is found that the phase profile of the out plane of the metasurface is more continuous than that of a three-level grating. The optical antenna can operate not only as a simple phase shift element, but also as an element to generate a more detailed phase profile. The particle swarm optimization (PSO) algorithm is applied. The numbers of particles and cycles are set as 20 and 1000, respectively. The diffractive efficiency can be optimized to 90% with the first coupling compensation (92% without the coupling compensation) with the design of optical antennas at rotation angles of 10.6°, 68.6°, and 130.0° (Figure 5b). Via the full metasurface simulation, the diffractive efficiency of the optimized three-phase meta-deflector is 86%. Although there is a small deviation (4%) by the coupling compensation method, the optimization tendencies are the same. Meanwhile, it is certain that the calculated DE is more accurate after the compensation. Furthermore, Figure 5d indicates that the gradient phase distribution is smoother than the distribution without the optimization.

## 4. Conclusions

In this paper, a new computational method is proposed to analyze the electromagnetic coupling of metasurface by coupling decomposition into different orders. The relationship between coupling factor and design parameters, including the period, length of antenna, wavelength, and polarization are analyzed. These parameters affect the intensity of the coupling, but the calculated field distribution is always convergent. It takes a long time to build a database of radiated and coupled field distribution for different parameters. To get the detailed field information with a small grid size, the simulation time becomes longer. On the other hand, this method fully supports the parallel computation, which can greatly increase the simulation speed. Because the decomposed radiated and coupled fields are independent. Furthermore, after building a database, the light distribution of the whole metasurface can be restructured rapidly and accurately. By PSO with the first-order coupling correction, the DE of a deflector based on the three-element metasurface can be optimized to 86%, which is much higher than theoretical DE for the three-phase deflector (~60%). It indicates that the light manipulation in metasurface design is not confined by the lattice of arrangement. The more detailed field distribution can also be controlled by proper design. This method can be expanded to arbitrary linear optical systems. If the processor, memory, and storage space of the computation workstation is large enough, the accuracy of the field restruction can be further improved. It has the potential to be applied to reduce the noise in the meta-hologram and optimize the metasurface with the relatively low refractive index dielectric material, such as glass, and metal metasurface with smaller feature sizes.
